# Beyond the Thyroid: A Narrative Review of Extra-thyroidal Manifestations in Hashimoto’s Disease

**DOI:** 10.7759/cureus.71126

**Published:** 2024-10-09

**Authors:** Palash S Kotak, Abhinav Kadam, Sourya Acharya, Sunil Kumar, Anuj Varma

**Affiliations:** 1 Internal Medicine, Jawaharlal Nehru Medical College, Datta Meghe Institute of Higher Medical Research, Wardha, IND

**Keywords:** autoimmune disorder, extra-thyroidal manifestations, hashimoto's disease, hypothyroidism, systemic involvement, thyroid dysfunction

## Abstract

Hashimoto's disease, the most common cause of hypothyroidism in iodine-sufficient regions, is traditionally viewed as a thyroid-specific autoimmune disorder. However, emerging evidence indicates that Hashimoto's disease has far-reaching systemic effects, manifesting in multiple organ systems beyond the thyroid gland. This comprehensive review aims to elucidate the extra-thyroidal manifestations of Hashimoto's disease, highlighting their pathophysiology, clinical presentation, and management strategies. The review explores neurological, cardiovascular, dermatological, gastrointestinal, musculoskeletal, and hematological manifestations, which can significantly impact the quality of life and complicate the clinical course of the disease. Neurological symptoms such as cognitive impairment, depression, and peripheral neuropathy, as well as cardiovascular complications like dyslipidemia and atherosclerosis, are increasingly recognized as significant concerns in patients with Hashimoto's disease. Additionally, autoimmune skin disorders, gastrointestinal motility issues, and musculoskeletal pain are discussed in the context of their connection to thyroid dysfunction. The review emphasizes the importance of recognizing these systemic manifestations for comprehensive patient management and suggests that a holistic approach, rather than focusing solely on thyroid hormone replacement, is essential. This review aims to improve diagnostic accuracy, treatment outcomes, and overall patient care by broadening the understanding of Hashimoto's disease to include its extra-thyroidal effects. Further research is encouraged to explore novel therapeutic approaches targeting the autoimmune mechanisms underlying these systemic manifestations.

## Introduction and background

Hashimoto's disease, also known as chronic lymphocytic thyroiditis, is a prevalent autoimmune disorder that primarily affects the thyroid gland [1]. First described by the Japanese physician Hakaru Hashimoto in 1912, it is now recognized as the leading cause of hypothyroidism in iodine-sufficient regions worldwide [2]. The disease occurs when the immune system mistakenly targets the thyroid gland, leading to chronic inflammation and progressive thyroid dysfunction. Affecting approximately 5-10% of the population, Hashimoto's disease is particularly common among women, especially those aged 30-50 years. It is also frequently seen in individuals with a family history of thyroid disorders or other autoimmune conditions [3].

The pathophysiology of Hashimoto's disease involves a complex interaction of genetic, environmental, and immunological factors. Central to the disease process is the infiltration of the thyroid gland by lymphocytes, predominantly T cells, which results in the formation of lymphoid follicles within the thyroid tissue [4]. Autoantibodies, such as thyroid peroxidase antibodies (TPOAb) and thyroglobulin antibodies (TgAb), are produced against thyroid-specific antigens, leading to the gradual destruction of thyroid cells and a decline in thyroid hormone production. While the exact triggers for this autoimmune response remain unclear, a combination of genetic predisposition, such as the presence of HLA-DR3 and HLA-DR5 genes, and environmental factors like iodine intake, infections, and stress are believed to contribute to the development of the disease [4].

Historically, clinical attention to Hashimoto's disease has been largely centered on the thyroid gland and its direct consequences. The hallmark symptoms, including fatigue, weight gain, cold intolerance, and dry skin, are primarily linked to hypothyroidism, the condition resulting from the decreased production of thyroid hormones (thyroxine and triiodothyronine) [1]. These hormones play a crucial role in regulating metabolism, and their deficiency leads to a wide array of physiological disturbances. Consequently, the management of Hashimoto's disease has traditionally focused on diagnosing hypothyroidism and administering thyroid hormone replacement therapy to restore normal metabolic function [1]. However, this narrow focus on thyroid-specific manifestations has led to an underappreciation of the broader, systemic effects that Hashimoto's disease can have. While the thyroid gland is the primary target, the autoimmune mechanisms underlying the disease can extend beyond the thyroid, affecting various organs and systems throughout the body [1].

This review aims to shift the clinical and research focus from the thyroid-centric view of Hashimoto's disease to a broader understanding that encompasses its extra-thyroidal manifestations. Recent studies have shed light on the fact that Hashimoto's disease can lead to a range of symptoms and complications affecting multiple organ systems, including the neurological, cardiovascular, dermatological, gastrointestinal, and musculoskeletal systems. Recognizing these systemic manifestations is crucial for comprehensive patient care. It allows healthcare providers to diagnose and address the full spectrum of the disease, potentially leading to earlier intervention and better outcomes. Moreover, understanding the systemic involvement in Hashimoto's disease may pave the way for novel therapeutic approaches that go beyond simple hormone replacement, focusing instead on modulating the underlying autoimmune processes.

## Review

Immunological and systemic involvement

HT is more than just a thyroid disorder; it often coexists with various autoimmune diseases and triggers systemic inflammatory responses. Understanding these associations provides valuable insights into the broader impact of HT on patient health [5]. HT is commonly linked with several other autoimmune diseases. Notably, there is a significant association between HT and celiac disease, especially in children and adolescents. The prevalence of celiac disease in patients with HT is considerably higher than in the general population, suggesting a shared autoimmune predisposition. Type 1 diabetes is among the most prevalent autoimmune comorbidities found in HT patients [6]. Studies indicate that approximately 18% of pediatric patients with HT also have type 1 diabetes, with a particularly pronounced association in younger populations and a higher frequency of autoimmune diseases observed in females compared to males [7]. HT can also co-occur with rheumatoid arthritis, an autoimmune condition characterized by joint inflammation. The presence of multiple autoimmune disorders in a single patient can complicate management and treatment strategies. Additional associated conditions include vitiligo, pernicious anemia, and Sjögren's syndrome. This clustering of diseases underscores the systemic nature of autoimmune responses in individuals with HT [8]. The systemic inflammatory response in HT involves various cytokines and immune cells that extend beyond the thyroid gland, affecting other organs and tissues. In HT, pro-inflammatory cytokines such as interleukin-6 (IL-6) and tumor necrosis factor-alpha (TNF-α) play significant roles in the inflammatory process [9]. These cytokines contribute to systemic inflammation, impacting the thyroid, other tissues, and organs, leading to various symptoms and complications. This systemic inflammatory response can lead to complications across various body systems [10]. For example, chronic inflammation may predispose patients to cardiovascular diseases, as elevated levels of thyroid autoantibodies have been linked to increased cardiovascular risk. Moreover, the inflammatory environment may affect the nervous system, potentially leading to cognitive impairments and other neurological symptoms [10].

Neurological and psychiatric manifestations

HT is not only marked by thyroid dysfunction but also has significant neurological and psychiatric manifestations. Among these, cognitive impairment and the commonly termed "brain fog" are prevalent in HT patients. The mechanisms underlying these cognitive deficits are complex. Hypothyroidism can cause changes in brain function due to a deficiency in thyroid hormones, which affects multiple cognitive domains, particularly memory, attention, and executive function [11]. Studies have shown that individuals with hypothyroidism often perform poorly on cognitive tasks, with memory being the most consistently affected area [12]. Additionally, neuroinflammation and neurotransmitter imbalances may contribute to cognitive impairment, as alterations in glutamate metabolism have been observed in the hippocampus, a brain region crucial for learning and memory. Furthermore, vitamin D deficiency, common in HT patients, has been associated with cognitive impairment, suggesting a potential neuroprotective role for this vitamin [12]. Clinically, cognitive impairment in HT patients typically manifests as difficulties with memory, concentration, and processing speed [13]. The Montreal Cognitive Assessment (MoCA) is commonly employed to evaluate cognitive function in these individuals. Management strategies often include thyroid hormone replacement therapy, such as levothyroxine, which can alleviate cognitive symptoms, although some deficits may persist despite treatment. Additionally, addressing vitamin D deficiency through supplementation may also prove beneficial in improving cognitive outcomes [13]. Another significant aspect of HT is its association with mood disorders, particularly depression and anxiety. There is a well-established link between thyroid function and mental health, with hypothyroidism increasing the risk of these mood disorders. The underlying mechanisms likely include hormonal influences, as thyroid hormones play a crucial role in regulating neurotransmitters like serotonin and norepinephrine, which are vital for mood stabilization. Moreover, the autoimmune nature of HT may contribute to neuroinflammation, further exacerbating mood disorders [14]. Studies indicate a higher prevalence of depression and anxiety in patients with HT compared to the general population. Treatment approaches for these conditions often involve psychotherapy, such as cognitive-behavioral therapy (CBT), and pharmacotherapy, where antidepressants may be prescribed alongside thyroid hormone replacement to address mood symptoms effectively [15]. In addition to cognitive and mood-related issues, other neurological manifestations of HT include peripheral neuropathy and myopathy. Peripheral neuropathy can present as numbness, tingling, and weakness in the extremities, with the exact mechanism not fully understood but likely related to autoimmune processes affecting nerve function [16]. Myopathy and muscle weakness are also common complaints among HT patients, often manifesting as generalized fatigue and decreased muscle strength. These symptoms may be attributed to the direct effects of thyroid hormone deficiency on muscle metabolism and function [16-21].

Dermatological manifestations

Dermatological features of Hashimoto’s disease can include dry skin, hair loss, and myxedema. Patients may also experience vitiligo or alopecia areata due to the autoimmune nature of the disease. Xerosis (dry skin) is particularly common and results from reduced eccrine gland function, while myxedema occurs due to the accumulation of glycosaminoglycans in the dermis [22].

Cardiovascular manifestations

Cardiovascular manifestations in HT are particularly significant when considering the impacts of dyslipidemia, hypertension, and related cardiovascular events. Understanding these connections is essential for effectively managing the overall health of individuals affected by this autoimmune disorder [23]. Hypothyroidism, a common outcome of HT, is closely associated with altered lipid metabolism. When thyroid hormone levels are low, there is a marked increase in low-density lipoprotein (LDL) cholesterol and total cholesterol levels, leading to dyslipidemia. This dyslipidemic profile poses a substantial risk for atherosclerosis, as elevated LDL cholesterol can promote the formation of atherosclerotic plaques within arterial walls [24]. Consequently, this increases the risk of cardiovascular disease (CVD) in patients with HT. Research shows that even mild hypothyroidism can predispose individuals to coronary artery disease. Furthermore, the presence of thyroid autoantibodies has been linked to a higher incidence of coronary artery disease, suggesting that autoimmune processes may further exacerbate cardiovascular risks. The combined effect of dyslipidemia and the inflammatory nature of HT significantly elevates the risk of cardiovascular morbidity and mortality [3]. Thyroid hormones play a critical role in regulating cardiovascular function, and their dysfunction can lead to various complications, including hypertension. In individuals with hypothyroidism, there is often an increase in systemic vascular resistance and a decrease in cardiac output, both of which contribute to elevated blood pressure [25]. The mechanisms underlying this include vascular reactivity and endothelial function alterations, which can exacerbate hypertension. Additionally, hypothyroidism is associated with changes in heart rate and contractility, further complicating the cardiovascular profile of affected individuals [25]. Numerous studies have demonstrated that individuals with thyroid dysfunction, particularly those with untreated hypothyroidism, face a heightened risk of cardiovascular events, including heart failure and myocardial infarction [26]. Evidence indicates that the risk of cardiovascular morbidity and mortality increases significantly when thyroid disorders are left untreated. However, restoring normal thyroid function through appropriate treatment often reverses these cardiovascular changes. This underscores the importance of monitoring and managing thyroid health in patients with HT to mitigate these significant cardiovascular risks effectively [25].

Gastrointestinal manifestations

HT can lead to a range of gastrointestinal manifestations, primarily driven by the associated hypothyroidism and autoimmune processes. This review examines the impact of HT on gastrointestinal motility disorders, autoimmune gastritis, and celiac disease [27]. Hypothyroidism, a frequent outcome of HT, significantly disrupts gastrointestinal motility. Patients often report symptoms such as slow gastric emptying, bloating, abdominal discomfort, and constipation. The underlying mechanism involves reduced thyroid hormone levels, essential for regulating gastrointestinal motility and digestive processes [28]. Thyroid hormones enhance the contractility of the gastrointestinal tract, and their deficiency can lead to decreased peristalsis, resulting in prolonged transit times and constipation. Studies have shown that individuals with hypothyroidism have a higher prevalence of functional gastrointestinal disorders, including constipation and irritable bowel syndrome (IBS) [29]. The pathophysiology of gastrointestinal motility disorders in hypothyroidism is multifaceted. Primarily, thyroid hormones are vital for stimulating gut motility; low levels can lead to diminished muscle contractions within the gastrointestinal tract [30]. Additionally, thyroid dysfunction can impair the autonomic nervous system, which regulates gut motility. Management strategies for these symptoms typically include thyroid hormone replacement therapy, which can alleviate gastrointestinal symptoms by normalizing thyroid hormone levels. Dietary modifications, such as increasing fiber intake and hydration, can help manage constipation, while medications like laxatives or prokinetic agents may be prescribed in more severe cases [30]. Autoimmune gastritis and celiac disease are gastrointestinal conditions that often co-occur with HT due to shared autoimmune mechanisms. Autoimmune gastritis involves the immune-mediated destruction of the gastric mucosa, leading to nausea, vomiting, and malabsorption [27]. Celiac disease is characterized by an immune response to gluten, resulting in inflammation and damage to the small intestine. Individuals with HT are at an increased risk of developing celiac disease, with studies indicating that up to 43% of patients may carry markers for this condition [27]. The clinical presentation of autoimmune gastritis typically includes nausea and vomiting, which stem from gastric inflammation and dysfunction, as well as malabsorption due to damage to the gastric mucosa and intestinal villi [31]. In celiac disease, symptoms can range from gastrointestinal issues like diarrhea and bloating to systemic manifestations such as fatigue and nutritional deficiencies. Diagnostic approaches for these conditions include serological testing to screen for specific antibodies, such as anti-tissue transglutaminase antibodies for celiac disease and endoscopy with biopsy to assess for mucosal damage in autoimmune gastritis and celiac disease. Given the overlap of these conditions with HT, healthcare providers should consider screening for celiac disease in patients with HT, especially when gastrointestinal symptoms are present [31].

Hematological manifestations

HT can lead to various hematological manifestations, particularly anemia coagulation and platelet function alterations. Understanding these complications is crucial for effective management and improving patient outcomes [32]. Anemia, especially iron deficiency anemia, is one of the most common hematological issues associated with HT. This condition can develop due to inadequate dietary intake, malabsorption, or chronic blood loss [33]. The prevalence of iron deficiency anemia among patients with hypothyroidism is notable, as hypothyroidism can impair iron metabolism and utilization. Moreover, pernicious anemia, an autoimmune disorder affecting vitamin B12 absorption, is frequently observed in patients with HT. The presence of intrinsic factor antibodies can hinder the absorption of vitamin B12, leading to megaloblastic anemia [33]. The diagnostic approach for anemia in patients with HT typically includes a complete blood count (CBC) to assess hemoglobin levels, red blood cell indices, and the presence of reticulocytes. Additional tests, such as serum ferritin and vitamin B12 levels, are necessary to determine the specific type of anemia. Detecting thyroid antibodies, including anti-TPO and anti-TG, can further support the diagnosis of HT [34]. Treatment for iron deficiency anemia generally involves oral iron supplementation, dietary modifications to increase iron intake, and addressing any underlying causes of blood loss. Vitamin B12 injections or high-dose oral B12 supplements are essential for pernicious anemia to bypass absorption issues. Additionally, thyroid hormone replacement therapy is critical for managing hypothyroidism and may help alleviate anemia symptoms [35]. Thyroid hormones play a significant role in hemostasis, and their deficiency can lead to coagulation and platelet function alterations. Hypothyroidism is associated with changes in the levels of various coagulation factors, including increased levels of factor VII, which can create a hypercoagulable state [36]. This heightened coagulation risk can elevate the likelihood of thromboembolic events in patients with HT. Beyond coagulation factors, thyroid hormones also influence platelet production and function. Hypothyroid patients may exhibit altered platelet aggregation and increased platelet counts, further contributing to thrombotic risks. These changes highlight the importance of monitoring coagulation parameters in patients with HT, as they may require tailored management strategies to mitigate potential complications [36].

Reproductive and endocrine manifestations

Thyroid dysfunction has a profound impact on reproductive health, influencing menstrual regularity, fertility, and interactions with other endocrine glands. Thyroid hormones are vital for the proper functioning of the female reproductive system, and both hypothyroidism and hyperthyroidism can cause significant menstrual disturbances. Hypothyroidism is often associated with oligomenorrhea (infrequent menstrual periods), while hyperthyroidism can lead to hypomenorrhea (light periods) and polymenorrhea (frequent periods) [37]. Infertility is another major consequence of thyroid dysfunction. In women, low levels of thyroid hormones can disrupt ovulation, a critical process for conception. Research indicates that thyroid disorders can contribute to subfertility or infertility by affecting ovarian, uterine, and placental function. Additionally, hypothyroidism has been linked to higher rates of miscarriage, preterm delivery, and low birth weight [38]. Managing thyroid dysfunction is essential for improving fertility outcomes. For women with hypothyroidism, it is crucial to ensure adequate thyroid hormone levels through replacement therapy before attempting to conceive. If infertility persists even after normalizing thyroid function, additional fertility treatments may be necessary [39]. In the case of hyperthyroidism, careful management is particularly important during pregnancy, as uncontrolled hyperthyroidism can lead to severe complications for both the mother and the baby. Fertility preservation strategies may involve monitoring and adjusting thyroid hormone levels, and in some cases, assisted reproductive technologies (ART) might be required [39]. Thyroid function is also closely linked with adrenal hormone activity. Hypothyroidism can coexist with adrenal insufficiency, which can compound the effects on reproductive health. The adrenal glands produce hormones essential for stress response and metabolic regulation, and their dysfunction can exacerbate symptoms associated with thyroid disorders [40]. The relationship between the thyroid and pituitary glands is crucial for maintaining hormonal balance. Thyroid hormones regulate the secretion of gonadotropins (LH and FSH) from the pituitary gland, which is critical for controlling the menstrual cycle and ovulation. Disruptions in thyroid function can lead to altered levels of these hormones, further affecting reproductive health [41].

Musculoskeletal manifestations

Hashimoto's thyroiditis, an autoimmune disorder that often leads to hypothyroidism, is associated with a variety of musculoskeletal manifestations, most notably arthralgia (joint pain) and myalgia (muscle pain). Understanding the underlying mechanisms of these symptoms and distinguishing them from other autoimmune conditions is essential for effective management [1]. Musculoskeletal pain in patients with Hashimoto's thyroiditis can be attributed to several mechanisms. A significant factor is autoimmune inflammation. The systemic inflammation associated with Hashimoto's can lead to joint and muscle pain, as elevated levels of inflammatory markers, such as cytokines, can heighten pain perception in affected individuals. Additionally, the direct effects of hypothyroidism contribute substantially to these symptoms [2]. Low thyroid hormone levels impair muscle strength, increase stiffness, and cause fatigue, all of which can manifest as muscle aches and joint discomfort. Hypothyroidism affects muscle metabolism and function, leading to myopathy (muscle disease) and increased pain sensitivity. Moreover, Hashimoto's thyroiditis frequently coexists with other autoimmune disorders, such as rheumatoid arthritis or fibromyalgia, which can complicate the clinical picture and contribute to musculoskeletal symptoms [42]. Differentiating musculoskeletal pain caused by Hashimoto's from pain caused by other autoimmune conditions requires a comprehensive clinical evaluation. A detailed patient history and thorough physical examination are essential for identifying specific patterns of joint involvement and associated symptoms that may point to other autoimmune diseases [43]. Laboratory tests for specific autoantibodies, such as rheumatoid factor, anti-cyclic citrullinated peptide (CCP), and inflammatory markers, can help differentiate Hashimoto's from other autoimmune disorders. For example, elevated thyroid peroxidase (TPO) antibodies are characteristic of Hashimoto's but do not indicate other autoimmune diseases. Imaging studies, such as X-rays or MRI, may be necessary to assess joint integrity and to rule out degenerative changes or other inflammatory arthritis conditions [43]. Hypothyroidism, particularly when associated with Hashimoto's thyroiditis, also has significant implications for bone health. Thyroid hormones are crucial for bone remodeling, and insufficient levels can lead to decreased bone density, which increases the risk of osteoporosis and fractures [44]. Studies have shown that patients with untreated or poorly managed hypothyroidism have a higher incidence of osteoporosis compared to the general population. Hypothyroidism can also negatively affect calcium and vitamin D metabolism, compromising bone health. Adequate levels of these nutrients are essential for maintaining bone density and preventing fractures [44]. To reduce the risk of osteoporosis and fractures in patients with Hashimoto's thyroiditis, several preventive measures and treatment strategies should be considered. The primary treatment for hypothyroidism is thyroid hormone replacement therapy, such as levothyroxine, which can help maintain bone health and reduce the risk of osteoporosis [45]. Ensuring sufficient calcium and vitamin D intake is also critical, and dietary modifications or supplementation may be necessary. Engaging in weight-bearing and resistance exercises is recommended to improve bone density and overall musculoskeletal health, as regular physical activity is crucial for preventing osteoporosis and maintaining muscle strength. Regular bone density screenings, particularly for older individuals or those with other risk factors, can help detect osteoporosis early and guide appropriate treatment decisions [45].

Ophthalmological manifestations

Ophthalmological manifestations in patients with Hashimoto's thyroiditis can significantly affect their quality of life, with key concerns including dry eyes, visual disturbances, and thyroid-related orbitopathy. Understanding these conditions is crucial for effective management and patient outcomes [46]. Hypothyroidism is closely associated with ocular surface disease (OSD), primarily characterized by dry eye syndrome (DES). Research shows that patients with thyroid dysfunction, including hypothyroidism, experience substantial ocular surface changes, with dry eye symptoms reported in 23% to 96% of cases [47]. Contributing factors include altered tear production and mechanical issues. The inflammatory process of thyroid disease can impact the lacrimal glands, leading to decreased tear production and increased evaporation. Additionally, eyelid position and proptosis changes can cause incomplete eyelid closure, worsening tear film instability, and corneal exposure [48]. Patients with ocular surface disease may present symptoms such as dryness, irritation, and visual disturbances. Clinical evaluation typically involves tear function tests, such as the Schirmer test and tear break-up time (TBUT), to assess tear production and stability [49]. Studies indicate that hypothyroid patients often have lower TBUT and Schirmer scores compared to healthy controls, reflecting compromised ocular surface health. Management strategies for dry eyes may include artificial tears, anti-inflammatory medications, and, in severe cases, punctal plugs to reduce tear drainage and improve moisture retention on the ocular surface [49]. Thyroid-related orbitopathy (TRO), also known as thyroid eye disease (TED), is a notable concern in patients with Hashimoto's thyroiditis, although it is more commonly associated with Graves' disease. The incidence of TRO in hypothyroid patients ranges from 0.2% to 8.6%. When it occurs, it can lead to serious ocular complications [50]. TRO is characterized by inflammation and swelling of the orbital tissues, resulting in symptoms such as proptosis, eyelid retraction, and visual disturbances. Proptosis, or forward displacement of the eye, results from swelling of the extraocular muscles and orbital fat, while increased palpebral fissure height can lead to ocular surface exposure and dry eyes [51]. Patients may also experience blurred vision or double vision due to muscle involvement. Management of TRO often includes a combination of medical and surgical treatments. Corticosteroids can effectively reduce inflammation in active cases, while surgical intervention may be required for severe proptosis or visual impairment. Orbital decompression surgery can relieve pressure on the optic nerve and improve ocular alignment [51].

Pediatric considerations

HT, though more common in adults, can also have significant effects on children and adolescents. The manifestations of this autoimmune disorder in the pediatric population present unique challenges that require careful attention in both diagnosis and management [52]. A primary concern for children with HT is growth retardation and delayed puberty. Hypothyroidism resulting from the condition can lead to stunted growth and delayed onset of secondary sexual characteristics, which is especially critical during the growth spurts of childhood and adolescence. These complications can affect a child's physical development if not promptly diagnosed and treated. Therefore, healthcare providers must vigilantly monitor growth patterns and address any abnormalities as they arise [53]. In addition to physical growth challenges, HT can impact cognitive and developmental aspects. Untreated hypothyroidism in children may result in significant cognitive deficits, affecting learning and overall development. Research highlights that thyroid hormones are crucial for brain development, particularly during early childhood. Consequently, early recognition and management of hypothyroidism are essential to mitigate the risk of long-term neurological consequences. Parents and educators should be aware of the potential for cognitive delays and advocate for appropriate evaluations and interventions when necessary [54]. Diagnosing and managing HT in pediatric patients can be particularly challenging due to differences in presentation compared to adults. Children may exhibit atypical symptoms or a more rapid disease progression, complicating the diagnostic process. This underscores the importance of maintaining a high index of suspicion among healthcare providers when evaluating pediatric patients with non-specific symptoms such as fatigue, weight changes, or mood disturbances. A thorough clinical evaluation and appropriate laboratory testing are critical for accurate diagnosis and timely treatment [53]. Treatment for HT in children requires careful consideration of age-appropriate dosing and monitoring. Pediatric patients may have different therapeutic needs than adults, and their treatment plans should be tailored to accommodate growth and developmental milestones. Regular follow-ups are essential to assess the effectiveness of therapy and make necessary adjustments. Consulting with pediatric endocrinologists or specialists experienced in managing autoimmune thyroid disorders is crucial for optimizing care [55]. Finally, the psychosocial impact of a chronic autoimmune condition like HT should not be overlooked. The diagnosis can be overwhelming for children and their families, leading to anxiety and stress. Providing support and education is vital to help them navigate the challenges associated with the disease and its treatment. Encouraging open communication and fostering a supportive environment can significantly enhance the overall well-being of affected individuals [56].

Diagnostic challenges and considerations

HT poses significant diagnostic challenges due to its varied clinical manifestations and potential overlap with other systemic diseases. A comprehensive clinical evaluation is essential for diagnosing HT, especially since its symptoms can be subtle and nonspecific [57]. Common manifestations include fatigue, weight gain, cold intolerance, and goiter. However, patients may also present with extra-thyroidal symptoms such as joint pain, cognitive impairment, and skin changes. These diverse presentations require a detailed patient history and physical examination to identify potential autoimmune manifestations beyond the thyroid [57]. Biomarkers are crucial in confirming the diagnosis of HT. The presence of thyroid peroxidase antibodies (TPOAbs) and thyroglobulin antibodies is typically indicative of the disease [58]. However, about 5-10% of patients may be seronegative, which can complicate the diagnosis. Imaging studies, particularly ultrasound, can provide additional insights into thyroid structure and function. Ultrasound can reveal characteristic features of HT, such as a heterogeneous gland and the presence of nodules, although it is not routinely necessary for diagnosis [58]. Differential diagnosis for HT includes various autoimmune and systemic diseases that may present with overlapping symptoms. Conditions such as chronic fatigue syndrome, fibromyalgia, and depression may be mistaken for HT due to similar fatigue and cognitive symptoms. Additionally, autoimmune diseases like lupus or rheumatoid arthritis may coexist with HT, further complicating the clinical picture. Careful assessment of the patient's symptoms and targeted laboratory tests are essential for differentiating HT from these other conditions [1]. Key diagnostic challenges and considerations in Hashimoto's disease are summarized in Table 1.

**Table 1:** Key diagnostic challenges and considerations in Hashimoto's disease TSH: thyroid stimulating hormone

Diagnostic Challenge	Description	Considerations	Impact on Diagnosis
Subclinical Hypothyroidism [59]	Early stages of Hashimoto’s often show normal thyroid hormone levels with elevated TSH.	Requires careful monitoring of TSH and thyroid antibodies.	May delay diagnosis due to normal hormone levels despite autoimmune activity.
Overlap with Other Autoimmune Diseases [60]	Hashimoto’s can coexist with other autoimmune conditions like celiac disease or lupus.	Screen for multiple autoimmune antibodies.	Coexisting conditions can obscure or complicate the diagnostic picture.
Non-specific Symptoms [61]	Fatigue, weight gain, and depression may mimic other conditions like chronic fatigue syndrome or depression.	Detailed patient history and exclusion of other conditions are crucial.	Non-specific symptoms often lead to misdiagnosis or delayed diagnosis.
Presence of Euthyroid Goiter [62]	Patients may present with thyroid enlargement but normal thyroid function.	Thyroid ultrasound and antibody testing can aid in diagnosis.	Goiter may be present without overt hypothyroidism, complicating diagnostic clarity.
Variation in Antibody Levels [63]	Thyroid peroxidase (TPO) antibodies may fluctuate, and thyroglobulin antibodies may not always be elevated.	Repeated antibody testing over time can help confirm the diagnosis.	Inconsistent antibody levels may confuse the diagnostic process.
Confounding Imaging Results [64]	Ultrasound findings such as hypoechogenicity may resemble other thyroid disorders like nodules or cancer.	Combine imaging with serological testing for accurate diagnosis.	Imaging alone may lead to misdiagnosis without complementary lab results.
Thyroid Nodule Presence [65]	Hashimoto’s patients can develop thyroid nodules, raising suspicion of malignancy.	A fine-needle aspiration biopsy may be required for nodule evaluation.	Nodules may lead to unnecessary biopsy or surgery if not carefully evaluated.

Management strategies

Managing extra-thyroidal manifestations of HT involves a blend of pharmacological and non-pharmacological strategies, underscoring the importance of multidisciplinary care. Long-term monitoring and follow-up are essential for evaluating extra-thyroidal involvement and adjusting treatment as needed [66]. The cornerstone of treatment for HT is lifelong thyroid hormone replacement with levothyroxine. This therapy aims to normalize thyroid hormone levels and alleviate symptoms of hypothyroidism, which can indirectly help manage some extra-thyroidal manifestations. In cases of significant inflammation, anti-inflammatory medications may be prescribed to alleviate symptoms such as joint pain and muscle aches [67]. For patients experiencing mood disorders like depression or anxiety, which are common in HT, antidepressants or anxiolytics may be beneficial. Additionally, if hormonal imbalances, such as menstrual irregularities, are identified, specific hormonal treatments may be initiated [14]. Non-pharmacological strategies play a critical role in managing HT. Patients are encouraged to follow a balanced diet rich in anti-inflammatory foods, engage in regular physical activity, and use stress management techniques like yoga or meditation. These lifestyle changes can enhance overall health and alleviate some symptoms associated with the condition [68]. Dietary adjustments, such as a gluten-free or anti-inflammatory diet, may relieve some patients, although these should be tailored to individual needs and sensitivities. Physical therapy can also benefit those with musculoskeletal symptoms, improving mobility and reducing pain [68]. A multidisciplinary approach is essential for the comprehensive management of HT. This often involves collaboration among endocrinologists, nutritionists, psychologists, and physical therapists. Such a collaborative care model ensures that thyroid and extra-thyroidal manifestations are managed holistically, addressing the patient's complex needs. Each specialist contributes expertise, leading to a more effective and personalized treatment plan [67]. Regular monitoring and long-term follow-up are critical. Patients should have thyroid function tests every six to 12 months to ensure that levothyroxine dosing is appropriate, with thyroid stimulating hormone (TSH) and T4 levels as key assessment markers. Ongoing evaluations should also include assessments of neurological, psychological, and musculoskeletal symptoms as well as any new or worsening manifestations. This may involve questionnaires or screenings for depression and cognitive function [69]. Given the association of HT with other autoimmune disorders, periodic screening for conditions such as celiac disease or rheumatoid arthritis may be warranted, especially if new symptoms arise. Patient education is also vital; individuals should be informed about the chronic nature of HT, the importance of adhering to treatment and follow-up appointments, and the need to report any new symptoms [1] promptly. Comprehensive management strategies for HT are detailed in Table 2.

**Table 2:** Comprehensive management strategies for Hashimoto's disease TSH: thyroid stimulating hormone

Management Strategy	Description	Indications	Considerations	Impact on Disease Management
Levothyroxine Replacement Therapy [70]	Synthetic thyroid hormone to restore normal thyroid function.	Indicated for patients with hypothyroidism or subclinical hypothyroidism.	Requires regular monitoring of TSH and free T4 levels to adjust the dose.	Effective in managing hypothyroid symptoms and stabilizing TSH levels.
Dietary Modifications [71]	Gluten-free, anti-inflammatory, or autoimmune protocols may be beneficial.	Recommended for patients with coexisting autoimmune diseases like celiac disease or those with inflammation.	May require consultation with a nutritionist for tailored dietary plans.	Can help reduce inflammation and support overall well-being.
Selenium Supplementation [72]	Selenium may reduce thyroid antibody levels and improve thyroid function.	Used in patients with elevated thyroid antibodies or mild thyroid dysfunction.	Should be administered cautiously to avoid selenium toxicity.	May help in reducing thyroid inflammation and progression of the disease.
Corticosteroids [73]	Occasionally used in cases of severe thyroiditis or inflammatory symptoms.	Reserved for patients with acute thyroiditis or severe inflammation.	Short-term use is recommended to avoid long-term side effects.	Can rapidly reduce inflammation but requires careful use due to side effects.
Monitoring and Observation [74]	Regular follow-up with thyroid function tests without immediate intervention.	Suitable for euthyroid patients or those with subclinical hypothyroidism.	Requires periodic assessment of thyroid function and antibody levels.	Helps in early identification of progression to overt hypothyroidism.
Surgical Intervention (Thyroidectomy) [75]	Removal of the thyroid gland in cases of large goiter, nodules, or suspicion of malignancy.	Indicated for patients with compressive symptoms or suspicious thyroid nodules.	Post-surgical hormone replacement therapy is necessary.	Eliminates large goiters or suspicious nodules but requires lifelong hormone replacement.
Stress Management and Lifestyle Modifications [76]	Implementing stress-reducing techniques such as yoga, meditation, and exercise.	Beneficial for patients experiencing stress-induced symptom flares.	Lifestyle modifications may need to be customized based on individual needs.	Helps in reducing symptom exacerbation and improves the quality of life.

Future directions and research

Recent research has significantly enhanced the understanding of HT pathophysiology. Key discoveries have emphasized the complex interplay between genetic and environmental factors, revealing that genetic predispositions and environmental triggers, such as stress and infections, play a crucial role in precipitating the autoimmune response [2]. Additionally, new insights into cytokine networks have shed light on the involvement of multiple cytokines in HT, demonstrating how these networks can exacerbate pro-inflammatory responses. This includes the activation of inflammasomes and defects in regulatory T cells, which may contribute to the loss of self-tolerance to thyroid autoantigens. Importantly, there is increasing evidence that the autoimmune process extends beyond the thyroid, influencing systemic inflammation and contributing to comorbidities such as cardiovascular diseases and other autoimmune disorders [77]. Innovative therapeutic approaches are being explored to address not only thyroid dysfunction but also the systemic effects of HT. Immunotherapies, such as Rituximab and Tocilizumab, are currently under investigation for their potential to target the autoimmune response effectively [5]. These treatments aim to suppress specific immune system components, thereby reducing thyroid damage and improving overall thyroid function. Additionally, novel combination therapies, such as LT4/T3 therapy and metformin, are being evaluated for their potential to enhance treatment efficacy and patient outcomes. These approaches may offer more tailored treatment options for patients who do not respond adequately to standard therapies. Research into selenium's role in thyroid hormone metabolism suggests that supplementation might help reduce antibody levels and improve thyroid function, although optimal dosing and long-term effects require further investigation [5]. Despite these advancements, several critical questions remain unanswered, underscoring the need for continued research in the field of HT. One significant area of inquiry is the mechanisms underlying autoimmunity; further research is needed to elucidate the precise pathways through which genetic and environmental factors trigger the autoimmune response. Understanding these mechanisms could lead to targeted preventive strategies. Additionally, as new treatments emerge, long-term studies are essential to assess their safety, efficacy, and impact on the quality of life for patients with HT [78]. This includes evaluating the potential of these therapies to mitigate systemic inflammation and associated comorbidities. Lastly, future research should focus on developing personalized treatment plans that account for individual patient characteristics, including genetic predispositions and specific immune profiles, to optimize therapeutic outcomes [78]. Future directions and emerging research in HT management are detailed in Table 3.

**Table 3:** Future directions and emerging research in Hashimoto's disease management

Area of Research	Description	Potential Impact	Current Challenges	Future Prospects
Personalized Medicine [79]	Developing individualized treatment plans based on genetic, environmental, and immunological factors.	Could optimize therapy and improve patient outcomes.	Requires extensive research on genetic markers and immune responses.	Potential for highly targeted therapies, improving efficacy, and minimizing side effects.
Immune Modulation Therapies [80]	Exploring therapies that modulate or suppress the immune response to slow disease progression.	May prevent or reverse autoimmune damage to the thyroid.	Safety concerns regarding long-term immune suppression.	Development of safer immune therapies that could halt or reverse autoimmune destruction.
Microbiome and Gut Health [81]	Investigating the role of gut microbiota in autoimmunity and Hashimoto’s progression.	May open new pathways for dietary or probiotic interventions.	Complex interactions between gut health and thyroid function need to be clarified.	Potential for dietary interventions or gut-targeted treatments to manage disease.
Biomarkers for Early Detection [82]	Identifying novel biomarkers to detect Hashimoto’s before clinical symptoms appear.	This could enable earlier diagnosis and intervention.	Lack of validated biomarkers for preclinical stages.	Improved screening and monitoring tools for at-risk individuals.
Thyroid Regeneration and Stem Cell Therapy [83]	Research into thyroid regeneration using stem cells or tissue engineering.	Could potentially restore normal thyroid function in affected patients.	Ethical and technical challenges in stem cell therapy development.	Future advancements may allow for thyroid tissue regeneration, reducing the need for lifelong hormone replacement.
Combination Therapies [84]	Studying the efficacy of combining levothyroxine with other treatments like selenium or probiotics.	May enhance treatment effectiveness and patient quality of life.	Need for more large-scale randomized controlled trials (RCTs).	Combination approaches may improve symptom control and disease progression.
Autoimmune Triggers Identification [2]	Identifying environmental, viral, or stress-related triggers that initiate Hashimoto’s disease.	This could lead to prevention strategies and early intervention.	Complex and multifactorial nature of autoimmune triggers.	Targeted prevention strategies based on trigger identification.

## Conclusions

In conclusion, Hashimoto's disease, traditionally viewed through the lens of thyroid dysfunction, encompasses a much broader spectrum of systemic effects that extend far beyond the thyroid gland. The autoimmune mechanisms driving this condition not only lead to hypothyroidism but also contribute to a variety of extra-thyroidal manifestations affecting multiple organ systems, including the neurological, cardiovascular, dermatological, gastrointestinal, and musculoskeletal systems. Recognizing these systemic involvements is critical for providing comprehensive care to patients, as it allows for earlier diagnosis, more effective treatment strategies, and better overall outcomes. By shifting the focus from a thyroid-centric perspective to a holistic understanding of Hashimoto's disease, clinicians and researchers can better address the full scope of the disease, ultimately improving patient management and quality of life. This expanded awareness is essential for the development of new therapeutic approaches that target not only the thyroid but also the underlying autoimmune processes affecting the entire body.

## References

[1] Mincer DL, Jialal I (2024). Hashimoto Thyroiditis. StatPearls [Internet].

[2] Franco JS, Amaya-Amaya J, Anaya JM (2013). Thyroid Disease and Autoimmune Diseases. Autoimmunity: From Bench to Bedside [Internet].

[3] Akamizu T, Amino N (2000). Hashimoto’s Thyroiditis. Endotext [Internet].

[4] Martínez-Hernández R, Sánchez de la Blanca N, Sacristán-Gómez P (2024). Unraveling the molecular architecture of autoimmune thyroid diseases at spatial resolution. Nat Commun.

[5] Bliddal S, Nielsen CH, Feldt-Rasmussen U (2017). Recent advances in understanding autoimmune thyroid disease: the tallest tree in the forest of polyautoimmunity. F1000Res.

[6] Ashok T, Patni N, Fatima M, Lamis A, Siddiqui SW (2022). Celiac disease and autoimmune thyroid disease: the two peas in a pod. Cureus.

[7] Hwang GB, Yoon JS, Park KJ, Lee HS, Hwang JS (2018). Prevalence of autoimmune thyroiditis in patients with type 1 diabetes: a long-term follow-up study. Ann Pediatr Endocrinol Metab.

[8] Cojocaru M, Cojocaru IM, Silosi I (2010). Multiple autoimmune syndrome. Maedica (Bucur).

[9] Wrońska K, Hałasa M, Szczuko M (2024). The role of the immune system in the course of Hashimoto’s thyroiditis: the current state of knowledge. Int J Mol Sci.

[10] Chen L, Deng H, Cui H (2018). Inflammatory responses and inflammation-associated diseases in organs. Oncotarget.

[11] Jansen HI, Boelen A, Heijboer AC, Bruinstroop E, Fliers E (2023). Hypothyroidism: the difficulty in attributing symptoms to their underlying cause. Front Endocrinol (Lausanne).

[12] Eslami-Amirabadi M, Sajjadi SA (2021). The relation between thyroid dysregulation and impaired cognition/behaviour: an integrative review. J Neuroendocrinol.

[13] Nasreddine ZS, Phillips NA, Bédirian V (2005). The Montreal Cognitive Assessment, MoCA: a brief screening tool for mild cognitive impairment. J Am Geriatr Soc.

[14] Nuguru SP, Rachakonda S, Sripathi S, Khan MI, Patel N, Meda RT (2022). Hypothyroidism and depression: a narrative review. Cureus.

[15] Parish AL, Gillis B, Anthamatten A (2023). Pharmacotherapy for depression and anxiety in the primary care setting. J Nurse Pract.

[16] (2024). Peripheral Neuropathy. https://www.ninds.nih.gov/health-information/disorders/peripheral-neuropathy.

[17] Samuels MH (2014). Psychiatric and cognitive manifestations of hypothyroidism. Curr Opin Endocrinol Diabetes Obes.

[18] Randhawa SS, Varghese D (2024). Geriatric Evaluation and Treatment of Age-Related Cognitive Decline. StatPearls [Internet].

[19] Hage MP, Azar ST (2012). The link between thyroid function and depression. J Thyroid Res.

[20] Beurel E, Toups M, Nemeroff CB (2020). The bidirectional relationship of depression and inflammation: double trouble. Neuron.

[21] Lin YJ, Kao TW, Chen WL (2021). Relationship between peripheral neuropathy and cognitive performance in the elderly population. Medicine (Baltimore).

[22] Cohen B, Cadesky A, Jaggi S (2023). Dermatologic manifestations of thyroid disease: a literature review. Front Endocrinol (Lausanne).

[23] Klubo-Gwiezdzinska J, Wartofsky L (2022). Hashimoto thyroiditis: an evidence-based guide to etiology, diagnosis and treatment. Pol Arch Intern Med.

[24] Mavromati M, Jornayvaz FR (2021). Hypothyroidism-associated dyslipidemia: potential molecular mechanisms leading to NAFLD. Int J Mol Sci.

[25] Yamakawa H, Kato TS, Noh JY, Yuasa S, Kawamura A, Fukuda K, Aizawa Y (2021). Thyroid hormone plays an important role in cardiac function: from bench to bedside. Front Physiol.

[26] Ning Y, Cheng YJ, Liu LJ (2017). What is the association of hypothyroidism with risks of cardiovascular events and mortality? A meta-analysis of 55 cohort studies involving 1,898,314 participants. BMC Med.

[27] Cellini M, Santaguida MG, Virili C, Capriello S, Brusca N, Gargano L, Centanni M (2017). Hashimoto’s thyroiditis and autoimmune gastritis. Front Endocrinol (Lausanne).

[28] Daher R, Yazbeck T, Jaoude JB, Abboud B (2009). Consequences of dysthyroidism on the digestive tract and viscera. World J Gastroenterol.

[29] Xu GM, Hu MX, Li SY, Ran X, Zhang H, Ding XF (2024). Thyroid disorders and gastrointestinal dysmotility: an old association. Front Physiol.

[30] Yaylali O, Kirac S, Yilmaz M, Akin F, Yuksel D, Demirkan N, Akdag B (2009). Does hypothyroidism affect gastrointestinal motility?. Gastroenterol Res Pract.

[31] Singh S, Chakole S, Agrawal S (2023). A comprehensive review of upper gastrointestinal symptom management in autoimmune gastritis: current insights and future directions. Cureus.

[32] Cao YT, Zhang KY, Sun J (2022). Platelet abnormalities in autoimmune thyroid diseases: a systematic review and meta-analysis. Front Immunol.

[33] Soliman AT, De Sanctis V, Yassin M, Wagdy M, Soliman N (2017). Chronic anemia and thyroid function. Acta Biomed.

[34] Turner J, Parsi M, Badireddy M (2024). Anemia. StatPearls [Internet].

[35] Warner MJ, Kamran MT (2024). Iron Deficiency Anemia. StatPearls [Internet].

[36] Elbers LP, Fliers E, Cannegieter SC (2018). The influence of thyroid function on the coagulation system and its clinical consequences. J Thromb Haemost.

[37] Koyyada A, Orsu P (2020). Role of hypothyroidism and associated pathways in pregnancy and infertility: clinical insights. Tzu Chi Med J.

[38] Cho MK (2015). Thyroid dysfunction and subfertility. Clin Exp Reprod Med.

[39] Mazzilli R, Medenica S, Di Tommaso AM (2023). The role of thyroid function in female and male infertility: a narrative review. J Endocrinol Invest.

[40] Wondisford FE (2015). A direct role for thyroid hormone in development of the adrenal cortex. Endocrinology.

[41] Brown ED, Obeng-Gyasi B, Hall JE, Shekhar S (2023). The thyroid hormone axis and female reproduction. Int J Mol Sci.

[42] Fariduddin MM, Haq N, Bansal N (2024). Hypothyroid Myopathy. StatPearls [Internet].

[43] Castro C, Gourley M (2010). Diagnostic testing and interpretation of tests for autoimmunity. J Allergy Clin Immunol.

[44] Williams GR, Bassett JH (2018). Thyroid diseases and bone health. J Endocrinol Invest.

[45] Apostu D, Lucaciu O, Oltean-Dan D, Mureșan AD, Moisescu-Pop C, Maxim A, Benea H (2020). The influence of thyroid pathology on osteoporosis and fracture risk: a review. Diagnostics (Basel).

[46] Kan E, Kılıçkan E, Ecemiş G, Beyazyildiz E, Çolak R (2014). Presence of dry eye in patients with Hashimoto’s thyroiditis. J Ophthalmol.

[47] Allam IY, Lazreg S, Shafik Shaheen M, Doheim MF, Mohammed MA (2021). Ocular surface changes in patients with thyroid eye disease: an observational clinical study. Clin Ophthalmol.

[48] Selter JH, Gire AI, Sikder S (2015). The relationship between Graves' ophthalmopathy and dry eye syndrome. Clin Ophthalmol.

[49] Messmer EM (2015). The pathophysiology, diagnosis, and treatment of dry eye disease. Dtsch Arztebl Int.

[50] Shah SS, Patel BC (2024). Thyroid Eye Disease. StatPearls [Internet].

[51] Şahlı E, Gündüz K (2017). Thyroid-associated ophthalmopathy. Turk J Ophthalmol.

[52] Kyritsi EM, Kanaka-Gantenbein C (2020). Autoimmune thyroid disease in specific genetic syndromes in childhood and adolescence. Front Endocrinol (Lausanne).

[53] Segni M (2000). Disorders of the Thyroid Gland in Infancy, Childhood and Adolescence. Endotext [Internet].

[54] Moog NK, Entringer S, Heim C, Wadhwa PD, Kathmann N, Buss C (2017). Influence of maternal thyroid hormones during gestation on fetal brain development. Neuroscience.

[55] Jonklaas J, Bianco AC, Bauer AJ (2014). Guidelines for the treatment of hypothyroidism: prepared by the american thyroid association task force on thyroid hormone replacement. Thyroid.

[56] Siegmann EM, Müller HH, Luecke C, Philipsen A, Kornhuber J, Grömer TW (2018). Association of depression and anxiety disorders with autoimmune thyroiditis. JAMA Psychiatry.

[57] Kolanu ND, Awan NA, Butt AI (2024). From antibodies to artificial intelligence: a comprehensive review of diagnostic challenges in hashimoto’s thyroiditis. Cureus.

[58] Fröhlich E, Wahl R (2017). Thyroid autoimmunity: role of anti-thyroid antibodies in thyroid and extra-thyroidal diseases. Front Immunol.

[59] Gosi SKY, Kaur J, Garla VV (2024). Subclinical Hypothyroidism. StatPearls [Internet].

[60] Boccuti V, Perrone A, D'Introno A, Campobasso A, Sangineto M, Sabbà C (2016). An unusual association of three autoimmune disorders: celiac disease, systemic lupus erythematosus and Hashimoto's thyroiditis. Auto Immun Highlights.

[61] Sapra A, Bhandari P (2024). Chronic Fatigue Syndrome. StatPearls [Internet].

[62] Führer D, Bockisch A, Schmid KW (2012). Euthyroid goiter with and without nodules--diagnosis and treatment. Dtsch Arztebl Int.

[63] (2024). Thyroid Antibodies Explained. https://www.btf-thyroid.org/thyroid-antibodies-explained.

[64] Wong KT, Ahuja AT (2005). Ultrasound of thyroid cancer. Cancer Imaging.

[65] Tamhane S, Gharib H (2016). Thyroid nodule update on diagnosis and management. Clin Diabetes Endocrinol.

[66] Haugen BR, Alexander EK, Bible KC (2016). 2015 American Thyroid Association Management Guidelines for adult patients with thyroid nodules and differentiated thyroid cancer: the American Thyroid Association Guidelines Task Force on thyroid nodules and differentiated thyroid cancer. Thyroid.

[67] Danailova Y, Velikova T, Nikolaev G, Mitova Z, Shinkov A, Gagov H, Konakchieva R (2022). Nutritional management of thyroiditis of Hashimoto. Int J Mol Sci.

[68] Osowiecka K, Myszkowska-Ryciak J (2023). The influence of nutritional intervention in the treatment of Hashimoto’s thyroiditis—a systematic review. Nutrients.

[69] Sheehan MT (2016). Biochemical testing of the thyroid: TSH is the best and, oftentimes, only test needed - a review for primary care. Clin Med Res.

[70] Antonelli A, Elia G, Ragusa F (2021). The stability of TSH, and thyroid hormones, in patients treated with tablet, or liquid levo-thyroxine. Front Endocrinol (Lausanne).

[71] Lerner A, Freire de Carvalho J, Kotrova A, Shoenfeld Y (2022). Gluten-free diet can ameliorate the symptoms of non-celiac autoimmune diseases. Nutr Rev.

[72] Ventura M, Melo M, Carrilho F (2017). Selenium and thyroid disease: from pathophysiology to treatment. Int J Endocrinol.

[73] Koirala KP, Sharma V (2015). Treatment of acute painful thyroiditis with low dose prednisolone: a study on patients from Western Nepal. J Clin Diagn Res.

[74] Villar HC, Saconato H, Valente O, Atallah AN (2007). Thyroid hormone replacement for subclinical hypothyroidism. Cochrane Database Syst Rev.

[75] Biello A, Kinberg EC, Wirtz ED (2024). Thyroidectomy. StatPearls [Internet].

[76] Lemay V, Hoolahan J, Buchanan A (2019). Impact of a yoga and meditation intervention on students’ stress and anxiety levels. Am J Pharm Educ.

[77] Ganesh BB, Bhattacharya P, Gopisetty A, Prabhakar BS (2011). Role of cytokines in the pathogenesis and suppression of thyroid autoimmunity. J Interferon Cytokine Res.

[78] Tomer Y (2014). Mechanisms of autoimmune thyroid diseases: from genetics to epigenetics. Annu Rev Pathol.

[79] Goetz LH, Schork NJ (2018). Personalized medicine: motivation, challenges, and progress. Fertil Steril.

[80] Rosenblum MD, Gratz IK, Paw JS, Abbas AK (2012). Treating human autoimmunity: current practice and future prospects. Sci Transl Med.

[81] Liu X, Liu J, Zhang T, Wang Q, Zhang H (2023). Complex relationship between gut microbiota and thyroid dysfunction: a bidirectional two-sample Mendelian randomization study. Front Endocrinol (Lausanne).

[82] Prasanth BK, Alkhowaiter S, Sawarkar G, Dharshini BD, R Baskaran A (2023). Unlocking early cancer detection: exploring biomarkers, circulating DNA, and innovative technological approaches. Cureus.

[83] Romitti M, Costagliola S (2023). Progress toward and challenges remaining for thyroid tissue regeneration. Endocrinology.

[84] Madan R, Celi FS (2020). Combination therapy for hypothyroidism: rationale, therapeutic goals, and design. Front Endocrinol (Lausanne).

